# The mediating role of psychological flexibility on the relationship between academic emotions, academic engagement, and academic achievement among university students

**DOI:** 10.3389/fpsyg.2025.1571717

**Published:** 2025-07-08

**Authors:** Ginesa López-Crespo, Goshgar Mursalzade, Noelia Sánchez-Pérez, Sonsoles Valdivia-Salas

**Affiliations:** Department of Psychology and Sociology, University of Zaragoza, Zaragoza, Spain

**Keywords:** psychological flexibility, academic engagement, academic emotions, academic achievement, mediation analysis

## Abstract

Previous literature has proven the positive relationship of academic emotions, academic engagement and academic achievement. However, less is known about the impact of what students do in response to this emotions - that is, their psychological flexibility- on academic engagement and achievement. Therefore, the main purpose of the present study was to investigate the role of psychological flexibility in the relationship between academic emotions, engagement and achievement in a sample of university students. With this purpose, 261 psychology students, aged 18 to 26, participated in a cross-sectional survey study. The results of the study revealed a significant mediator role of psychological flexibility between the academic emotions of enjoyment and study academic achievement. Specifically, enjoyment was related to academic achievement both directly and indirectly through psychological flexibility. In contrast, study boredom did not have a direct relationship with academic achievement, but an indirect relationship mediated both by psychological flexibility and by the influence of psychological flexibility on engagement. These findings highlight the importance of psychological flexibility in the academic environment, showing its potential to help students to improve their academic performance regardless of the valence of the emotion experienced during their studies.

## Introduction

Academic studies are a long-distance run, and students encounter numerous frustrations and academic adversities along the way. Therefore, in recent decades, the importance of emotions in achieving academic success has been widely emphasized (see for example, Camacho-Morles et al., 2021). Emotions are regarded as a complex phenomenon that encompasses various dimensions, including cognitive, physiological, affective, motivational, and expressive aspects (Scherer, 2001). It has been observed that when performing academic tasks like completing homework, assignments or taking exams, students encounter a variety of emotions such as enjoyment, hope, pride, relief, anger, anxiety, shame, hopelessness, and boredom (Pekrun et al., 2002a).

The definition of these specific emotions is crucial as they affect students’ learning and academic achievement, both in general and in university students. Indeed, a recent systematic review indicates that academic performance is positively associated with positive valence emotions such as enjoyment and negatively associated with negative emotions such as anger and boredom (Camacho-Morles et al., 2021). One of the mechanisms that have been proposed to explain this relationship is student engagement (e.g., Pekrun and Linnenbrink-Garcia, 2012). Generally speaking, engagement refers to the level of students’ commitment to their tasks and activities (for a review, see Upadyaya and Salmela-Aro, 2013), and there is evidence of its linking role between positive emotions and improved academic achievement (Carmona-Halty et al., 2021).

From this point of view, to improve academic performance, we should encourage positive valence emotions and minimize negative ones, which will enhance engagement and subsequently academic achievement (Carmona-Halty et al., 2021). However, recent perspectives in the study of emotion suggest that it is not just the emotion we experience but rather our responses to that emotion what can significantly impact the results we obtain. The underlying construct behind this is psychological flexibility, which is regarded as the ability to pursue what is important while staying in contact with current thoughts and emotions, particularly the unwanted ones (Hayes et al., 2006; Kashdan and Rottenberg, 2010). In an educational context, students with high psychological flexibility can experience various emotions while still pursuing their long-term goals. Therefore, psychological flexibility could be a key variable to understand the connection between academic emotions and other academic outcomes, including engagement and academic achievement.

## Literature review

Research exploring the role of psychological flexibility within educational settings is relatively recent. A systematic review conducted in our laboratory (Mursalzade et al., 2025) demonstrated that psychological flexibility in higher education students is positively associated with psychological adjustment and negatively associated with maladjustment. Regarding academic outcomes, this review found the field to be in an incipient stage of development. Nevertheless, it highlighted promising studies linking psychological flexibility with variables closely related to academic success, such as self-regulated learning (Asikainen, 2018), self-efficacy (Ateş and Sağar, 2024; Jeffords et al., 2020), college adjustment (Bi and Li, 2021), self-efficacy and achievement goals (Martinie and Shankland, 2024), procrastination (Hailikari et al., 2021) and burn-out (Asikainen and Katajavuori, 2023). Furthermore, from an interventional perspective, studies have shown that increasing psychological flexibility through Acceptance and Commitment Therapy (ACT) can improve not only psychological health but also academic outcomes in university students (Asale et al., 2021; Gregoire et al., 2016; Grégoire et al., 2018; Katajavuori et al., 2023, 2025; Räihä et al., 2024; Viskovich and Pakenham, 2018).

Regarding the variables outlined in the introduction, psychological flexibility has been directly associated with academic emotions (Asikainen et al., 2018; Hailikari et al., 2022), academic engagement (Martinie and Shankland, 2024), and academic performance (Asikainen et al., 2018; Ateş and Sağar, 2024; Hailikari et al., 2022). Moreover, several studies have advanced this understanding by showing that psychological flexibility can mediate the relationship between two of these variables–academic emotions and achievement. This aligns with perspectives from authors who argue that the connection between emotions and achievement is not a direct one, but instead is mediated by various other processes (Pekrun, 2006, 2024; Mega et al., 2014; Tze et al., 2016; Valiente et al., 2012). In this sense, Sandoz et al. (2017) found a mediational role of psychological flexibility in the relationship between statistic anxiety and performance. Similar results were observed in another specific domain, namely music. In this regard, Viator et al. (2024) discovered that psychological flexibility is related to both music performance anxiety and students’ perceived performance. Consistent with this, Doğan’s (2024) study found a mediation relationship between test anxiety, rumination, and psychological flexibility, although it did not investigate how these interactions influenced academic performance. The mediational role of psychological flexibility was also addressed by Daşcı et al. (2023), who found that psychological flexibility was negatively related to intolerance of uncertainty but positively related to some measures of academic adjustment as perception of success, educational stress and academic self-efficacy, but surprisingly, not to academic achievement itself. In contrast, Asikainen et al. (2018) finding that psychological flexibility mediated the relationship between positive and negative academic emotions and both study pace and academic success. In a subsequent work, Hailikari et al. (2022) further explored the interrelationship among psychological flexibility, academic emotions, and study pace/academic success, finding a complex relationship between all of them. This study also revealed that students with high, medium, and low psychological flexibility scores differed significantly in their academic emotions, as well as in their study pace and academic success. However, despite some studies suggesting that engagement also mediates between academic emotions and academic performance (Carmona-Halty et al., 2021; Oriol-Granado et al., 2017; Pekrun and Linnenbrink-Garcia, 2012), and as noted earlier, research linking psychological flexibility and engagement Martinie and Shankland (2024), to our knowledge, no study to date has comprehensively examined the serial interrelationship among psychological flexibility, academic emotions, academic engagement, and academic success.

## Objectives

**Figure 1:**
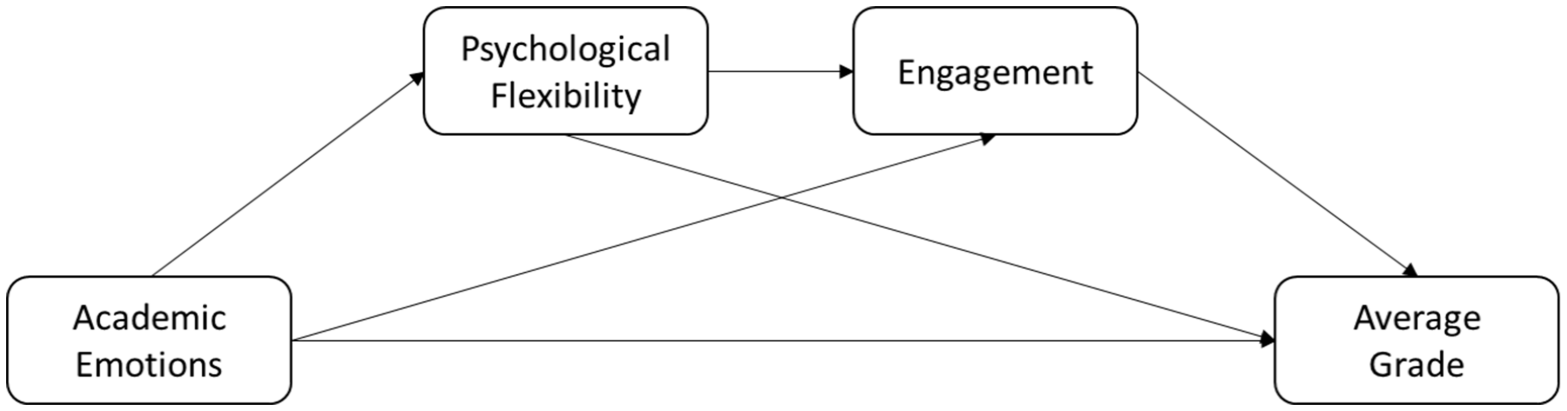
Proposed model for the relationship between academic emotions, psychological flexibility, engagement and average grade.

This study aims to address the previously mentioned gap by investigating the relationships between academic emotions and academic achievement within a sample of university students. Specifically, we aimed to examine the mediating role of psychological flexibility and academic engagement between different academic emotions and academic achievement (see Figure 1).

## Methodology

### Participants

The study involves 261 psychology degree students from a public university located in Spain. The sampling system used was convenience, the most used in this type of study (Etikan et al., 2016). Table 1 summarizes the primary sociodemographic characteristics of the sample. The age range comprised from 18 to 26 years, with an average of 19.79 years (SD = 1.620). Informed consent was obtained from all participants prior to their involvement in the study.

**Table 1:** Participant sociodemographic characteristics.

	Sociodemographic information	*N* (%)
Gender	Male	42 (16.10%)
	Female	203 (77.80%)
	Not specified	16 (6.10%)
Academic year	First year	81 (31.0%)
	Second year	74 (28.4%)
	Third year	43 (16.5%)
	Fourth year	63 (24.1%)

### Instruments

To measure academic engagement, we used the Utrecht Work Engagement Scale for Students (UWES-S) (Schaufeli et al., 2006). The Spanish adaptation of the well-known questionnaire consisted of 15 items that obtained their score using a Likert scale of 7 points (from 0, never, to 6, every day) adapted to the academic context (López-Crespo et al., 2022) was used. Although the original scale was conformed of three subscales (dedication, vigor and absorption), preliminary analyses conducted in our laboratory revealed reliability problems with the absorption subconstruct (Cronbach alphas between 0.52 and 0.57; unpublished data), and hence, we eliminated the absorption subscale. Therefore, the final version of the scale was composed of 10 items belonging to the vigor subscale (5 items) and dedication subscale (5 items). Examples of items were: “I can study for long periods of time” (vigor) and “My studies are inspiring” (dedication). The reliability analyses showed that the vigor scale had an alfa of Chronbach of 0.80 and 0.90 for the dedication scale. The total reliability of the scale was of 0.89.

To measure psychological flexibility, the Work-related Acceptance and Action Questionnaire (WAAQ) (Bond et al., 2013) was employed. It consists of a scale made up of seven items that are scored using a seven-point Likert scale (from 1, never true to 7, always true). For this study, the Spanish validation carried out by Ruiz and Odriozola-Gonzalez (2014) was used and adapted to the academic context. Examples of items were: “I can study effectively even when I am nervous for some reason.” The scale showed a high reliability (alfa of Chronbach of 0.90) in our study.

Academic emotions were measured by the Achievement Emotions Questionnaire (AEQ) scale (AEQ; Pekrun et al., 2002a), adapted to the Spanish (Sánchez-Rosas, 2015). Although the original AEQ addresses activity emotions (enjoyment, boredom, and anger), prospective outcome emotions (hope, anxiety, and hopelessness), and retrospective outcome emotions (pride, relief, and shame), for the sake of brevity, we included only shame, class boredom, study boredom and enjoyment. A 5-point Likert scale (1 = completely disagree, 5 = completely agree) was used to record item responses. Example of items were “I enjoy being in class” (enjoyment), “In class, I feel embarrassed” (shame), “I get bored in class” (class boredom) and “Studying is monotonous and boring” (study boredom). The reliability analysis results revealed the highest Chronbach’s alpha value for the Shame subscale (0.92), while the lowest reliability was recorded for the Class boredom subscale (0.73). The value of Chronbach’s alpha for in Enjoyment (0.88) and study Boredom (0.87) subscales were also high.

Finally, academic performance was measured using the students’ average grade (GPA) from first-semester exam results. This unit of measurement is the most commonly used in studies of these characteristics, as, to date, none has shown a better ability to quantify academic performance (Richardson et al., 2012).

### Statistical analysis

For the treatment and analysis of the data, the statistical data analysis package SPSS Statistics 19, from IBM, was used. First, a reliability analysis was conducted to measure the accuracy of the constructs employed in the study. Next, correlation analyses, including main and control (age, school year) variables, were conducted. Finally, serial mediation analyses were conducted to investigate the mediating role of psychological flexibility and engagement between academic emotions and GPA. The PROCESS macro developed by Hayes (2017) was employed to conduct the mediational analyses.

## Results

### Correlation analysis

Table 2 show the results of the descriptive and correlation analyses performed on the scores of the above mentioned questionnaires and GPA. The results revealed that GPA correlated low but significantly with psychological flexibility, vigor, dedication and enjoyment but not with the other academic emotions examined (shame, class boredom and study boredom). Psychological flexibility correlated low or moderately but significantly with all the examined variables, except class boredom. A significant moderate positive correlation relationship was also revealed between dedication and vigor subscales of academic engagement scale and GPA. Both variables also correlated significantly with all the examined variables, at low or moderate levels. Regarding academic emotions, they correlated with each other at low or moderate levels, except shame and enjoyment and shame and class boredom. The direction of all correlations was as expected: positive for all the variables except those correlations involving negative emotions, which were negative (see Table 2).

**Table 2:** Descriptive and correlation analysis results.

	Mean ± SD	1	2	3	4	5	6	7	8
1. GPA	6.20 ± 1.5								
2. Psy. flexibility	27.78 ± 7.41	0.269^**^							
3. Dedication	21.01 ± 5.33	0.174^**^	0.291^**^						
4. Vigor	15.12 ± 4.93	0.250^**^	0.420^**^	0.612^**^					
5. Enjoyment	24.52 ± 5.18	0.242^**^	0.150^*^	0.580^**^	0.487^**^				
6. Shame	21.32 ± 8.53	−0.003	−0.227^**^	−0.137^*^	−0.123^*^	0.028			
7. Class boredom	23.30 ± 6.37	−0.114	−0.118	−0.313^**^	−0.319^**^	−0.527^**^	0.014		
8. Study boredom	26.44 ± 6.75	- 0.063	−0.285^**^	−0.499^**^	−0.533^**^	−0.376^**^	0.171^**^	0.539^**^	

*p < 0.05; ** *p* < 0.01.

Additional correlational analyses were conducted to see if age, year of studies and GPA correlated to each other. The results showed that GPA was significantly and positively related to both year of study (*r* = 0.522, *p* < 0.001) and age (*r* = 0.302, *p* < 0.001).

### Mediation analysis results

To investigate whether academic emotions influence GPA both directly and indirectly, a sequential mediation analysis was conducted following the schema presented in Figure 1. Specifically, this analysis focused on the emotions of enjoyment and study boredom, as these opposite valence emotions correlated with the most variables to a greater extent both in our study and in others (Camacho-Morles et al., 2021). Furthermore, since the two engagement scales correlated to each other, only the total engagement score was considered in these analyses. Finally, as both year of career and age influenced GPA, their effects were controlled by including these variables as covariates in the mediational analyses.

#### Positive valence emotion: enjoyment

**Figure 2:**
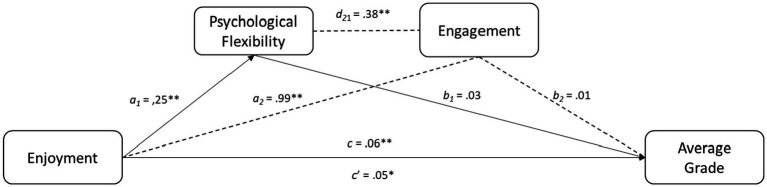
Result of the mediational analysis. Continuous lines represent significant direct and indirect effects. Discontinuous lines represent non significative indirect effects; * *p* < 0.05; ** *p* < 0.01.

Enjoyment had a direct effect on GPA [B = 0.05, SE = 0.02; 95% CI (0.01, 0.08), *p* < 0.05]. In addition, it had an indirect effect on GPA through psychological flexibility [B = 0.01, SE = 0.01; 95% CI (0.00, 0.02)]. No other indirect effect was statistically significant (see Figure 2).

#### Negative valence emotion: study boredom

**Figure 3:**
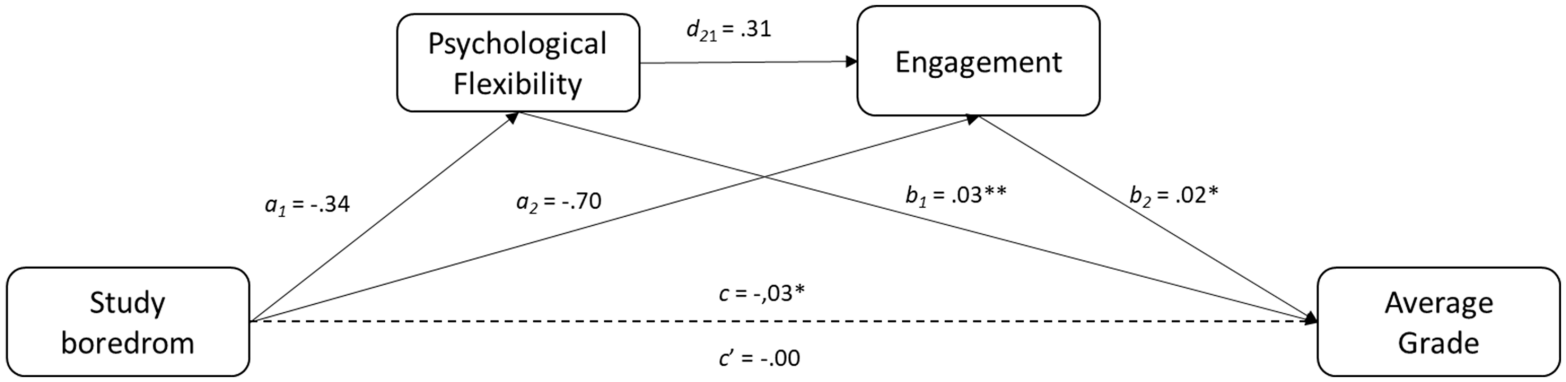
Result of the mediational analysis. Continuous lines represent significant indirect effects. Discontinuous lines represent non significative direct and indirect effects; * *p* < 0.05; ** *p* < 0.01.

Study boredom did not directly influence GPA. However, indirectly it influenced GPA throughout both psychological flexibility [B = −0.01, SE = 0.00; 95% CI (−0.02, −0.00), *p* < 0.01] and engagement [B = −0.01, SE = 0.01; 95% CI (−0.03, −0.00), *p* < 0.01]. Also, GPA was indirectly influenced by boredom through the sequential indirect effect of psychological flexibility on engagement [B = 0.00, SE = 0.00; 95% CI (−0.01, −0.00), *p* < 0.01] (see Figure 3).

## Discussion

The main aim of the present study was to examine the role of psychological flexibility and academic engagement as mediators in the relation between academic emotions, such as enjoyment and boredom, and academic achievement.

In line with previous research (Hayat et al., 2017; Villavicencio and Bernardo, 2013), we found a direct positive relationship between enjoyment and participants’ GPA. According to Pekrun et al. (2002b) enjoyment is one of the several emotions experienced in educational settings and has been associated with the use of deeper and more comprehensive learning strategies (Pekrun and Hofmann, 1999), more stable and enduring effort (Gendolla, 2003), higher self-regulation (Villavicencio and Bernardo, 2013) and higher engagement (Reschly et al., 2008). Finding a direct association with GPA, hence, adds to this evidence and emphasizes the importance of finding ways to make university studies an exciting and enjoyable experience.

Besides the direct benefits of enjoyment on GPA, we also found indirect benefits through the mediation of psychological flexibility. It seems that enjoyment facilitates the flexible coping with unwanted feelings and emotions typically associated to academic routines (e.g., deadlines, assignments, lack of sleep, etc.). Similar results had been reported by Asikainen et al. (2018) regarding the emotion of hope, using the accumulation of ECTS credits as a proxy for study progression. In her study, psychological flexibility had a greater impact on study progression than self-regulation, climate or commitment to studies. Our study’s findings align with these results, demonstrating that psychological flexibility impacts academic achievement.

In the case of boredom, we only found an indirect negative relation with GPA through the mediation of psychological flexibility according with Pekrun (2006), boredom compromises students’ emotional and behavioral processes, increasing the likelihood of avoiding academic routines. This is precisely what we observed in our study: boredom seems to hinder the flexible coping with unwanted thoughts and feelings. Interestingly, we also found an indirect positive effect of psychological flexibility on GPA through the mediation of an increased engagement. That is, the student’s ability to be open to their internal unpleasant processes appears to increase dedication and vigour, which, in turn, seems to improve academic performance. In line with this idea, previous research indicates that students who score high in psychological flexibility tend to persist and achieve their academic goals despite facing various challenges (Kashdan and Rottenberg, 2010).

Considering both findings together, it seems that regardless of the valence of the current emotion (yet enjoyment, yet boredom), relating to such emotion with flexibility has beneficial effects. Enjoyment appears to facilitate psychological flexibility, whereas boredom seems to hinder its use, but once psychological flexibility is activated (with less or more difficulties), engagement and GPA improve.

### Limitations

Despite our novel findings, the study has several limitations. First, the article employed non-experimental research methods. For deeper insights, conducting an experimental study would be more suitable. Secondly, the sample size is relatively small. More accurate results could be obtained with a larger sample size. In addition, as these findings were obtained within a specific sociocultural context, caution should be exercised regarding their generalizability to other populations or settings. Lastly, the transversal nature of the design employed does not allow to draw firm conclusions about the direction of the relations observed. Longitudinal studies controlling for a larger number of variables are necessary.

## Conclusion

Studies investigating psychological flexibility as a mediator between various academic variables, including academic achievement and academic engagement, are rare. In this context, our study gives a step forward, showing a preponderant role of psychological flexibility since it systematically mediated the relationship between different emotions, both positive (enjoyment) and negative (study boredom), and academic achievement. This mediational relationship is both direct and indirect through its influence on engagement in case of study boredom. These findings offer promising insights, yet future research should aim to replicate them in diverse sociocultural contexts and expand the range of academic emotions investigated. Furthermore, exploring the specific facets of psychological flexibility (e.g., cognitive defusion) that drive these relationships will be crucial. A deeper understanding of these mechanisms will enable the development of more precise and effective interventions.

## Data Availability

The raw data supporting the conclusions of this article will be made available by the authors, without undue reservation.

## References

[ref1] Asale M. Ayalew S. Kibret B. (2021). Enhancing academic adjustment, motivation and life satisfaction of female preparatory students using acceptance and commitment therapy. Bahir Dar J. Educ. 21, 1–21. doi: 10.4314/bdje.v21i2

[ref9001] Asikainen H. (2018). Examining indicators for effective studying–The interplay between student integration, psychological flexibility and self-regulation in learning. Psychol. Soc. Educ. 10, 225–237. doi: 10.25115/psye.v10i2.1873

[ref2] Asikainen H. Hailikari T. Mattsson M. (2018). The interplay between academic emotions, psychological flexibility and self-regulation as predictors of academic achievement. J. Further High. Educ. 42, 439–453. doi: 10.1080/0309877X.2017.1281889

[ref3] Asikainen H. Katajavuori N. (2023). Exhausting and difficult or easy: the association between psychological flexibility and study related burnout and experiences of studying during the pandemic. Front. Educ. 8:1215549. doi: 10.3389/feduc.2023.1215549

[ref4] Ateş B. Sağar M. E. (2024). Investigation of academic success, psychological flexibility and self-efficacy in teacher candidates. Lang. Teach. Educ. Res. 7, 14–23. doi: 10.35207/later.1292374

[ref5] Bi D. Li X. (2021). Psychological flexibility profiles, college adjustment, and subjective well-being among college students in China: a latent profile analysis. J. Context. Behav. Sci. 20, 20–26. doi: 10.1016/j.jcbs.2021.01.008

[ref6] Bond F. W. Lloyd J. Guenole N. (2013). The work-related acceptance and action questionnaire: initial psychometric findings and their implications for measuring psychological flexibility in specific contexts. J. Occup. Organ. Psychol. 86, 331–347. doi: 10.1111/joop.12001

[ref7] Camacho-Morles J. Slemp G. R. Pekrun R. Loderer K. Hou H. Oades L. G. (2021). Activity achievement emotions and academic performance: a meta-analysis. Educ. Psychol. Rev. 33, 1051–1095. doi: 10.1007/s10648-020-09585-3

[ref8] Carmona-Halty M. Salanova M. Llorens S. Schaufeli W. B. (2021). Linking positive emotions and academic performance: the mediated role of academic psychological capital and academic engagement. Curr. Psychol. 40, 2938–2947. doi: 10.1007/s12144-019-00227-8

[ref9] Daşcı E. Salihoğlu K. Daşcı E. (2023). The relationship between tolerance for uncertainty and academic adjustment: the mediating role of students’ psychological flexibility during COVID-19. Front. Psychol. 14:1272205. doi: 10.3389/fpsyg.2023.1272205, PMID: 38046112 PMC10691738

[ref10] Doğan U. (2024). The relationship between test anxiety, rumination, and psychological flexibility. Curr. Psychol. 43, 2568–2577. doi: 10.1007/s12144-023-04411-9

[ref11] Etikan I. Musa S. Alkassim R. (2016). Comparison of convenience sampling and purposive sampling. Am. J. Theor. Appl. Stat. 5, 1–4. doi: 10.11648/j.ajtas.20160501.11

[ref12] Francis A. W. Dawson D. L. Golijani-Moghaddam N. (2016). The development and validation of the comprehensive assessment of acceptance and commitment therapy processes (CompACT). J. Context. Behav. Sci. 5, 134–145. doi: 10.1016/j.jcbs.2016.05.003

[ref13] Gendolla G. H. E. (2003). “Mood effects and effort mobilisation in learning: theory and experimental evidence” in Learning emotions: the influence of affective factors on classroom learning. eds. Mayring P. H. von Rhoeneck C. H. (Frankfurt, Germany: Peter Lang), 29–46.

[ref14] Grégoire S. Lachance L. Bouffard T. Dionne F. (2018). The use of acceptance and commitment therapy to promote mental health and school engagement in university students: a multisite randomized controlled trial. Behav. Ther. 49, 360–372. doi: 10.1016/j.beth.2017.10.003, PMID: 29704966

[ref15] Gregoire S. Lachance L. Bouffard T. Hontoy L. M. De Mondehare L. (2016). The effectiveness of the approach of acceptance and commitment with regard to the psychological health and academic engagement of university students. Can. J. Behav. Sci. 48, 222–231.

[ref16] Hailikari T. Katajavuori N. Asikainen H. (2021). Understanding procrastination: a case of a study skills course. Soc. Psychol. Educ. 24, 589–606. doi: 10.1007/s11218-021-09621-2

[ref17] Hailikari T. Nieminen J. Asikainen H. (2022). The ability of psychological flexibility to predict study success and its relations to cognitive attributional strategies and academic emotions. Educ. Psychol. 42, 626–643. doi: 10.1080/01443410.2022.2059652

[ref18] Hayat A. A. Esmi K. Rezaei R. Nabiee P. (2017). The relationship between academic emotions and academic performance of medical students of Shiraz University of Medical Sciences. Res. Med. Educ. 9, 29–20.

[ref19] Hayes A. F. (2017). Introduction to mediation, moderation, and conditional process analysis: a regression-based approach. New York: Guilford publications.

[ref20] Hayes S. C. Luoma J. B. Bond F. W. Masuda A. Lillis J. (2006). Acceptance and commitment therapy: model, processes and outcomes. Behav. Res. Ther. 44, 1–25. doi: 10.1016/j.brat.2005.06.006, PMID: 16300724

[ref21] Jeffords J. R. Bayly B. L. Bumpus M. F. Hill L. G. (2020). Investigating the relationship between university students’ psychological flexibility and college self-efficacy. J. Coll. Stud. Retent. Res. Theory Pract. 22, 351–372. doi: 10.1177/1521025117751071, PMID: 33867862 PMC8049598

[ref22] Kashdan T. B. Rottenberg J. (2010). Psychological flexibility as a fundamental aspect of health. Clin. Psychol. Rev. 30, 865–878. doi: 10.1016/j.cpr.2010.03.001, PMID: 21151705 PMC2998793

[ref23] Katajavuori N. Hailikari T. Asikainen H. (2025). Enhancing students’ well-being and studying in higher education: a comparison of two different study skill courses. Front. Educ. 10:1485784. doi: 10.3389/feduc.2025.1485784

[ref24] Katajavuori N. Vehkalahti K. Asikainen H. (2023). Promoting university students’ well-being and studying with an acceptance and commitment therapy (ACT)-based intervention. Curr. Psychol. 42, 4900–4912. doi: 10.1007/s12144-021-01837-x

[ref25] Li Y. Bebiroglu N. Phelps E. Lerner R. M. Lerner J. V. (2008). Out-of-school time activity participation, school engagement and positive youth development: findings from the 4-H study of positive youth development. J. Youth Dev. 3:22. doi: 10.5195/jyd.2008.284

[ref26] López-Crespo G. Blanco-Gandía M. C. Valdivia-Salas S. Fidalgo C. Sánchez-Pérez N. (2022). The educational e-portfolio: preliminary evidence of its relationship with student’s self-efficacy and engagement. Educ. Inf. Technol. 27:1. doi: 10.1007/s10639-021-10827-2

[ref27] Martinie M. A. Shankland R. (2024). Achievement goals, self-efficacy, and psychological flexibility as antecedent of study engagement. Soc. Psychol. Educ. 27, 1–22. doi: 10.1007/s11218-024-09921-3

[ref28] Mega C. Ronconi L. De Beni R. (2014). What makes a good student? How emotions, self-regulated learning, and motivation contribute to academic achievement. J. Educ. Psychol. 106:121. doi: 10.1037/a0033546

[ref29] Mursalzade G. Escriche-Martínez S. Valdivia-Salas S. Jiménez T. I. López-Crespo G. (2025). Factors associated with psychological flexibility in higher education students: a systematic review. Sustain. For. 17:5557. doi: 10.3390/su17125557

[ref30] Oriol-Granado X. Mendoza-Lira M. Covarrubias-Apablaza C. G. Molina-López V. M. (2017). Positive emotions, autonomy support and academic performance of university students: the mediating role of academic engagement and self-efficacy. Rev. Psicodidact. 22, 45–53. doi: 10.1387/RevPsicodidact.14280

[ref31] Pekrun R. (2006). The control-value theory of achievement emotions: assumptions, corollaries, and implications for educational research and practice. Educ. Psychol. Rev. 18, 315–341. doi: 10.1007/s10648-006-9029-9

[ref32] Pekrun R. (2024). Control-value theory: from achievement emotion to a general theory of human emotions. Educ. Psychol. Rev. 36:83. doi: 10.1007/s10648-024-09909-7

[ref33] Pekrun R. Goetz T. Titz W. Perry R. P. (2002a). Academic emotions in students' self-regulated learning and achievement: a program of qualitative and quantitative research. Educ. Psychol. 37, 91–105. doi: 10.1207/S15326985EP3702_4

[ref34] Pekrun R. Goetz T. Titz W. Perry R. (2002b). “Positive emotions in education” in Beyond coping: meeting goals, visions and challenges in beyond coping: meeting goals, visions, and challenges. ed. Frydenberg E.. (Oxford: Oxford Academic), 149–174.

[ref35] Pekrun R. Hofmann H. (1999). “Lern- und Leistungsemotionen: Erste Befunde eines Forschungsprogramms. [learning and achievement emotions. First results of a research program]” in Emotion, motivation und Leistung [emotion, motivation, and achievement]. eds. Jerusalem M. Pekrun R. (Göttingen: Hogrefe), 247–267.

[ref36] Pekrun R. Linnenbrink-Garcia L. (2012). “Academic emotions and student engagement” in Handbook of research on student engagement. eds. Reschly A. L. Christenson S. L. (Boston, MA: Springer), 259–282.

[ref37] Räihä K. Katajavuori N. Vehkalahti K. Asikainen H. (2024). Effects of study-integrated well-being course intervention for different burnout and engagement profiles of university students. Front. Learn. Res. 12, 45–68. doi: 10.14786/flr.v12i3.1415

[ref38] Reschly A. L. Huebner E. S. Appleton J. J. Antaramian S. (2008). Engagement as flourishing: the contribution of positive emotions and coping to adolescents’ engagement at school and with learning. Psychol. Sch. 45, 419–431. doi: 10.1002/pits.20306

[ref39] Richardson M. Abraham C. Bond R. (2012). Psychological correlates of university students' academic perfomance: a systematic review and meta-analysis. Psychol. Bull. 138, 353–387. doi: 10.1037/a0026838 22352812

[ref40] Ruiz F. Odriozola-Gonzalez P. (2014). The Spanish version of the work-related acceptance and action questionnaire (WAAQ). Psicothema 26, 63–68. doi: 10.7334/psicothema2013.221 24444731

[ref41] Sánchez-Rosas J. (2015). The achievement emotions questionnaire-argentine (AEQ-AR): internal and external validity, reliability, gender differences and norm-referenced interpretation of test scores. Rev. Evaluar 15, 41–74. doi: 10.35670/1667-4545.v15.n1.14908

[ref42] Sandoz E. K. Butcher G. Protti T. A. (2017). A preliminary examination of willingness and importance as moderators of the relationship between statistics anxiety and performance. J. Context. Behav. Sci. 6, 47–52. doi: 10.1016/j.jcbs.2017.02.002

[ref43] Schaufeli W. Bakker A. Salanova M. (2006). The measurement of work engagement with a short questionnaire. Educ. Psychol. Meas. 66, 701–716. doi: 10.1177/00131644052824

[ref44] Scherer K. R. (2001). “Appraisal considered as a process of multilevel sequential checking” in Appraisal processes in emotion. eds. Scherer K. R. Schorr A. Johnstone T. (Oxford, England: Oxford University Press), 92–120.

[ref45] Tze V. M. Daniels L. M. Klassen R. M. (2016). Evaluating the relationship between boredom and academic outcomes: a meta-analysis. Educ. Psychol. Rev. 28, 119–144. doi: 10.1007/s10648-015-9301-y

[ref46] Upadyaya K. Salmela-Aro K. (2013). Development of school engagement in association with academic success and well-being in varying social contexts: a review of empirical research. Eur. Psychol. 18:136. doi: 10.1027/1016-9040/a000143

[ref47] Valiente C. Swanson J. Eisenberg N. (2012). Linking students’ emotions and academic achievement: when and why emotions matter. Child Dev. Perspect. 6, 129–135. doi: 10.1111/j.1750-8606.2011.00192.x, PMID: 23115577 PMC3482624

[ref48] Viator T. L. Levy J. J. Murphy B. A. (2024). Exploring the relations among psychological flexibility, music performance anxiety, and perceived performance quality with university music students. Music Sci. 7:20592043241268665. doi: 10.1177/20592043241268665

[ref49] Villavicencio F. T. Bernardo A. B. (2013). Positive academic emotions moderate the relationship between self-regulation and academic achievement. Br. J. Educ. Psychol. 83, 329–340. doi: 10.1111/j.2044-8279.2012.02064.x, PMID: 23692538

[ref50] Viskovich S. Pakenham K. I. (2018). Pilot evaluation of a web-based acceptance and commitment therapy program to promote mental health skills in university students. J. Clin. Psychol. 74, 2047–2069. doi: 10.1002/jclp.22656, PMID: 29962090

